# Interactions of free-living amoebae with rice bacterial pathogens *Xanthomonas oryzae* pathovars *oryzae* and *oryzicola*

**DOI:** 10.1371/journal.pone.0202941

**Published:** 2018-08-24

**Authors:** John J. Long, Courtney E. Jahn, Andrea Sánchez-Hidalgo, William Wheat, Mary Jackson, Mercedes Gonzalez-Juarrero, Jan E. Leach

**Affiliations:** 1 Department of Bioagricultural Sciences and Pest Management, Colorado State University, Fort Collins, Colorado, United States of America; 2 Mycobacteria Research Laboratories, Department of Microbiology, Immunology, and Pathology, Colorado State University, Fort Collins, Colorado, United States of America; Fujian Agriculture and Forestry University, CHINA

## Abstract

**Background:**

Free-living amoebae (FLA) are voracious feeders, consuming bacteria and other microbes during colonization of the phytobiome. FLA are also known to secrete bacteriocidal or bacteriostatic compounds into their growth environment.

**Methodology and principal findings:**

Here, we explore the impacts of co-cultivation of five FLA species, including *Acanthamoeba castellanii*, *A*. *lenticulata*, *A*. *polyphaga*, *Dictyostelium discoideum* and *Vermamoeba vermiformis*, on survival of two devastating bacterial pathogens of rice, *Xanthomonas oryzae* pathovars (pv.) *oryzae* and *oryzicola*. In co-cultivation assays, the five FLA species were either bacteriostatic or bactericidal to *X*. *oryzae* pv. *oryzae* and *X*. *oryzae* pv. *oryzicola*. Despite these effects, bacteria were rarely detected inside amoebal cells. Furthermore, amoebae did not disrupt *X*. *oryzae* biofilms. The bactericidal effects persisted when bacteria were added to a cell-free supernatant from amoebal cultures, suggesting some amoebae produce an extracellular bactericidal compound.

**Conclusions/Significance:**

This work establishes novel, basal dynamics between important plant pathogenic bacteria and diverse amoebae, and lays the framework for future mechanistic studies.

## Introduction

Free-living amoebae (FLA) are ubiquitous microorganisms found in soil and water across the globe. Amoebae live a predominantly heterotrophic lifestyle–preying on a variety of other microorganisms such as bacteria, fungi and even other protists. Amoebae can directly and indirectly impact plants. For example, *Acanthamoeba castellanii*, when added to the rhizospheres of rice or *Arabidopsis thaliana*, markedly changes the plants’ microbiome composition and root architecture, and increases dry biomass [1, 2]. Amoebae also influence plant development, likely through increases in the soil nitrogen pool [3].

Bacteria-amoebae dynamics are complex and nuanced. Amoebae can greatly alter bacterial community composition and structure due to their prolific grazing and prey selectivity [4, 5]. Not all bacterial species are preyed upon equally, and bacteria have evolved a variety of defense mechanisms to deter amoebal feeding. Some bacteria use biofilms to shield themselves from feeding, while others directly lyse amoeba, either after internalization or through secretion of toxic factors [6–8]. Other bacterial species prevent lysis after phagocytosis, and, in fact, exploit the amoeba as a reservoir or host [9–11].

Amoebae-bacteria interactions have been described for diverse combinations, including interactions with both animal and plant bacterial pathogens [11–16]. In cases where amoebae detrimentally affect the bacteria, it may be possible to exploit that relationship to reduce bacterial loads in a system. For example, use of amoebae to reduce populations of plant pathogenic bacteria has been proposed as a form of biocontrol [17–19]. However, due to the interaction specificity of the amoebae-bacteria combination and the large effort required to describe those interactions, general themes have not yet been identified.

Bacterial pathogens of major importance but with no described interactions with free-living amoebae are the rice (*Oryza sativa*) pathogens within the species *Xanthomonas oryzae*. *X*. *oryzae* are gram-negative bacteria and the causative agents of two distinct and severe diseases of rice. *X*. *oryzae* pathovar (pv.) *oryzae* colonizes plant vasculature and causes bacterial blight while pv. *oryzicola* inhabits the spaces between mesophyll cells and leads to bacterial leaf streak [20]. Both pathovars are of major concern to rice production, commonly responsible for the loss of 10 to 20% of infected crops. In severe outbreaks and during the monsoon season, an entire crop may be lost [21]. This devastation is a salient issue as rice is one of the most important food crops, feeding over half of the world’s population and a majority of the impoverished [22]. Due to its impact on a staple crop, *X*. *oryzae* is regulated by extensive international quarantines and is designated as a Select Agent by the U.S. Animal and Plant Health Inspection Services and the Centers for Disease Control [23]. The most common method to minimize disease impacts is the deployment of resistant rice varieties. However, resistant varieties have only been developed against *X*. *oryzae* pv. *oryzae*, and this pathogen readily evolves to overcome host resistance mechanisms [24–26]. To date, affordable, effective and sustainable chemical controls are not available [27]. As such, alternative strategies to control the diseases are needed.

There are several reasons to consider the interface between amoebae and *X*. *oryzae* for pathogen control. Both organisms are found in soil, plant debris or on the surfaces of plants [4, 14, 28, 29], thus it is plausible that there is contact between the amoebae and the bacteria. As detailed above, there are several possible scenarios for outcomes following amoebae-bacteria contact, but these are not known for *X*. *oryzae–*amoeba interactions. Previous work showed that populations of a closely related bacteria, *X*. *campestris*, decline when the bacteria are inoculated into soil occupied by protozoa [30], but the authors did not identify which protozoa or other soil factors were responsible for the effect. Given amoebal dietary preferences and the various strategies bacteria use to avoid predation by amoebae, our goal was to characterize the interactions of five different amoebae, *Acanthamoeba polyphaga*, *A*. *lenticulata*, *A*. *castellanii*, *Vermamoeba vermiformis* and *Dictyostelium discoideum* with the two pathovars of *X*. *oryzae*. Based on interactions with other bacteria, we hypothesized that the interactions would be antagonistic, with bacterial numbers being reduced in the presence of amoebae. However, our studies demonstrated that the tested amoebae were bactericidal or bacteriostatic, depending on the amoebal species, and that some amoebal cell-free supernatants were lethal to *X*. *oryzae*.

## Materials and methods

### Amoebae and *X*. *oryzae* culturing

*A*. *polyphaga* Linc-AP1, *A*. *castellanii* ATCC 30234, and *A*. *lenticulata* ATCC 30841 were cultured at 28°C in a modified PYG medium (ATCC medium 712, pH of 6.9). *V*. *vermiformis* ATCC 50237 was cultured at 28 °C in a modified PYNFH medium (ATCC medium 1034, pH of 6.4) and *D*. *discoideum* ATCC NC4A1:DBS0236602 was maintained at room temperature in a modified HL5 medium (pH 6.7); media were modified according to Wheat et al. [31]. Amoeba cultures were inoculated into 100 x 15 mm petri dishes with 30 mm walls holding 10 mL of media supplemented with 1x Gibco penicillin/streptomycin (Invitrogen; Carlsbad, CA, USA) from frozen stocks. Once initial cultures reached turbidity, *Acanthamoeba* species and *V*. *vermiformis* were passaged every 5 days by transferring 500 μL of culture into 10 mL of fresh medium. *D*. *discoideum* were passaged every 3 days. Amoebal cultures were used for only three passages before disposal.

*X*. *oryzae* pvs. *oryzae* (strain PXO99^A^) and *oryzicola* (strain BLS256) were maintained on peptone sucrose agar medium (PSA; 10 g/L Bacto peptone, 10 g/L sucrose, 1 g/L monosodium glutamate, and ± 16 g/L Bacto agar) at 28 °C. Bacteria were streaked onto PSA from frozen glycerol stocks and cultured for two to three days before use.

### Co-culture kinetics

Confluent cultures of amoebae were starved overnight in diluted medium at the temperatures described above, except for *D*. *discoideum*, which did not survive incubation in the diluted medium. *Acanthamoeba* were starved at 1/5 strength PYG diluted with Page’s modified Neff amoeba saline (PAS) while *V*. *vermiformis* was starved in 1/2 PYNFH medim diluted with PS broth [31]. Amoebal cell density was calculated using a direct cell counting method involving trypan blue exclusion and a hemocytometer. Only cultures consisting of at least 90% viable trophozoites were used. Amoebae cultures were adjusted to concentrations of 2×10^5^ cells/mL.

*X*. *oryzae* cells were suspended into 1X phosphate buffered saline (PBS; 8.006 g/L NaCl, 0.2013 g/L KCl, 1.4916 g/L Na_2_HPO_4_, and 0.245 g/L KH_2_PO_4_) and washed three times with centrifugations at 2500 x G for 5 min to remove extracellular polysaccharide and biofilm. After washing, cells were suspended to an OD600 of 0.2, roughly 3×10^7^ CFU/mL.

Amoebae and *X*. *oryzae* co-cultures were prepared in 1.5 mL microcentrifuge tubes at an amoebae-to-bacteria cell ratio of 1:10. The final volume of co-cultures per tube equaled 500 μL with approximately 1×10^5^ trophozoites and 1×10^6^ CFU bacteria. Controls with a single species in the starvation medium were included and triplicates of each combination were prepared for processing at 0, 4, and 24 h. At the designated sampling times, cultures were spun at 150 x G for 2 min to pellet amoeba. The supernatant was removed for viable cell count assays of *X*. *oryzae*. The amoebal pellet was washed once with 1 mL of PAS, centrifuged at 150 x G for 2 min, then suspended in 500 μL PAS supplemented with 30 μg/mL gentamicin for 1 h to lyse any extracellular *X*. *oryzae* adhering to the amoebae [13]. The amoebal pellet was then washed three times in the conditions described above, and then suspended in 200 μL of PAS. Amoebae were disrupted by passaging through a 27-gauge syringe seven times and lysate was used in viable cell count assays of *X*. *oryzae* internalized inside amoebae [32].

Co-culture supernatants and amoeba lysates were assayed for live *X*. *oryzae* by ten-fold serial dilutions down to 1×10^−6^. 10 μL of the original fraction and all dilutions were plated on PS agar in technical triplicates, and, after two days at 28 °C, the bacterial colonies were counted.

### Internalization assays

Amoebae and bacterial cultures were prepared similarly to the kinetic assays. Additionally, *X*. *oryzae* cells were stained with the LIVE/DEAD BacLight cell viability kit (Invitrogen L7007) following removal of biofilm. Co-cultures were prepared in the same manner as described above and samples were processed at 0, 4, and 24 h. Cultures were spun at 150 x G for 2 min and supernatant was discarded. The amoebal pellet was treated with 100 μg/mL gentamicin, and after 1 h, washed three times. Cells were fixed using 100 μL 4% paraformaldehyde (w/v; dissolved in PBS). Immediately after fixative addition, 25 μL aliquots of each replicate were pooled together and 75 μL 0.4% trypan blue was added to determine viability of amoebae. Samples were fixed for 48 h at 4 °C in the dark, then washed once and suspended in 100 μL PAS. Fixed samples were stored up to 2 weeks at 4 °C in the dark.

Amoebae were imaged for internalized *X*. *oryzae* on a Zeiss LSM510 inverted confocal laser scanning microscope. Samples were excited with a 488 nm laser and emission filters were set to 480 nm and 590 nm for Syto9 and propidium iodide, respectively. At a 630x magnification, three random fields were taken per sample and images were taken at ten different depths in 0.5–1.5 μm increments. Images were compiled into one using the Zeiss Zen 2009 software. Total amoebae, number of encysted amoebae, and the number of amoebae with internalized fluorescent signals were recorded.

Samples fixed concurrently with trypan blue were imaged on a Zeiss Axioskop light microscope. Samples were added to a hemocytometer and counted for number of live and dead amoebae.

### Conditioned media assays

Amoebae culture densities were calculated using a hemocytometer and 0.4% trypan blue and cultures were adjusted to 5×10^4^ cells/mL. Amoebae cultures were aliquoted into 15 mL conical tubes with 5 mL of culture in each, one conical tube held an aliquot at 5×10^5^ cells/mL for the high-density amoebae-only conditioning treatment. Amoebae aliquots were spun at 200 x G for 5 min and the supernatant was discarded. Cells were suspended in 1X culturing medium, except for *V*. *vermiformis*, which was suspended in a 1:1 mix of PYNFH and PS broth.

Washed *X*. *oryzae* pv. *oryzae* was added to one tube of amoebae at an amoebae-to-bacteria ratio of 1:10 (approximately 5×10^5^ CFU). *X*. *oryzae* pv. *oryzicola* was added in the same manner to another aliquot of amoebae. The four conditioning cultures (low and high-density amoebae-only and two amoebae + *X*. *oryzae*) were incubated at 28 °C, except for *D*. *discoideum*, which was incubated at room temperature. After 48 h the cultures were spun at 1000 x G for 5 min. The supernatant from each was passed through a cellulose acetate syringe filter with 0.22 μm pores (VWR #28145–477; Randor, PA, USA). An aliquot of each was tested for pH levels to record any changes after conditioning.

Fresh *X*. *oryzae* cells were washed three times in 1X PBS to remove biofilm and finally resuspended to an OD600 of 0.1. 10 μL of *X*. *oryzae* and 190 μL of conditioned medium were seeded into a 96-well microplate. High-density conditioning medium supplemented with fresh medium were mixed in a 1:1 ratio prior to addition and diluted, fresh medium controls were included. Samples were prepared in triplicate for sampling at each time point at 4 and 24 h, two samples of each pathovar in fresh medium and in HD conditioned medium were sampled at 0 h to establish initial bacterial density. Microplates were incubated at 28 °C and samples were diluted and plated for viable bacteria as described in the co-culture kinetics.

### Crystal violet staining of biofilms

Suspensions of both *X*. *oryzae* pathovars were adjusted to OD600 of 0.5; bacteria were not washed in order to retain their biofilm. Aliquots (150 μL of each pathovar) were added to an untreated, polystyrene 96-well microplate (Corning #3370) in replicates of 24. The plate was enclosed in a sealable plastic bag and incubated at 28 °C for 24 h to allow bacteria to form biofilms.

Amoebae were starved overnight in diluted medium as performed for the co-culture kinetics (except for *D*. *discoideum*). Amoebae cell density was calculated using 0.4% trypan blue and a hemocytometer. Aliquots of amoebae cultures were generated at concentrations of 1×10^5^, 1×10^3^, and 1×10^1^ cells/mL by spinning and resuspending in fresh starvation medium. *X*. *oryzae* liquid cultures were removed from the microplate, leaving the ring-shaped biofilm and associated bacteria. 200 μL of each amoebae culture was added to the biofilms in replicates of six, and fresh medium controls were added as well. The microplate was sealed in a plastic bag and returned to a 28 °C incubator.

Biofilms were exposed to amoebae for 24 or 48 h, at which point an adapted crystal violet staining method was applied to quantify remaining biofilm [33]. Briefly, after removal of liquid cultures and rinsing with 200 μL of distilled water, the biofilm was treated with 200 μL of 0.5% crystal violet (w/v; dissolved in 10% ethanol) per well. After 15 min, crystal violet was removed, the wells were rinsed once with 150 μL of distilled water, and the remaining crystal violet was dissolved with 200 μL of 90% ethanol. The microplate was agitated at medium intensity for 2 min on a Biotek Powerwave HT plate reader, and absorbance at 570 nm was recorded.

## Results and discussion

### *X*. *oryzae* survival is reduced in the presence of amoeba trophozoites

**Fig 1:**
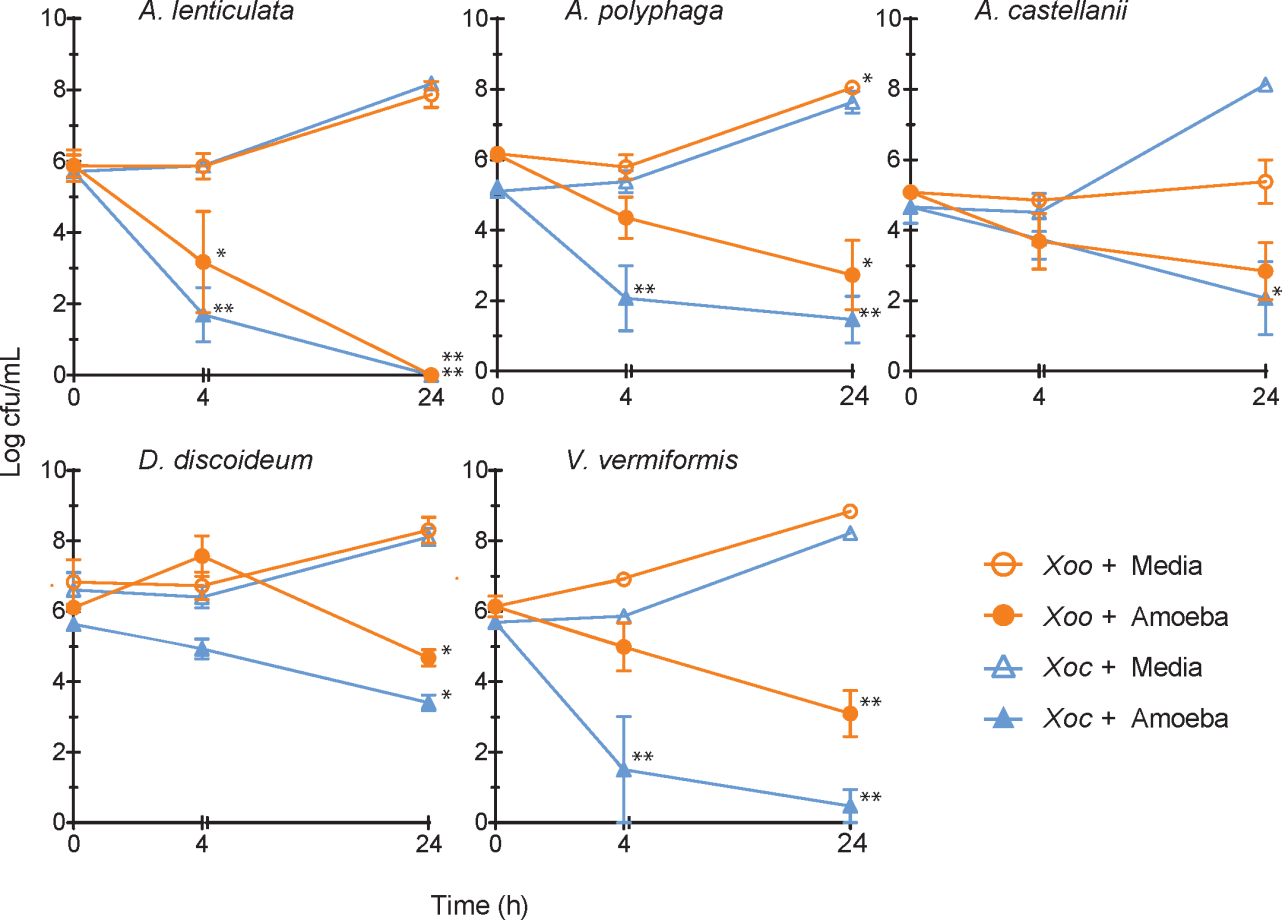
Populations of *X*. *oryzae* pvs. *oryzae* and *oryzicola* decline or remain static when co-cultured with amoeba trophozoites. Trophozoites were cultured with *X*. *oryzae* cells and bacterial numbers in the co-culture medium were assessed at 0, 4 and 24 h. Data from at least four biological replicates were log transformed before calculating the means and standard error bars. * denotes p < 0.05 and ** denotes a p < 0.01 compared to initial CFU/mL values at 0 h. Statistical significance tested using a two-way ANOVA (Tukey test) on the log-transformed data.

Amoebae trophozoites and *X*. *oryzae* bacterial cells were co-cultivated to determine the impact on bacterial populations, amoebal morphology and survival over time. Amoebae species, except *D*. *discoideum*, were incubated in diluted medium overnight prior to co-cultivation to encourage phagocytosis. *D*. *discoideum* was incubated in full strength medium prior to co-cultivation because it did not survive starvation induced by diluted media. Nine of the ten amoeba-*X*. *oryzae* combinations resulted in significant growth disparities over time (Fig 1). *A*. *lenticulata*, *A*. *polyphaga*, *V*. *vermiformis* and *D*. *discoideum* were bactericidal, with significant reductions in bacterial numbers (CFU) after 24 h (Tukey’s test, p < 0.05). *A*. *lenticulata* and *V*. *vermiformis* displayed the strongest effects against the bacteria, as bacteria were undetectable at 24 h in some cases. The rate of bacterial cell death varied between amoeba species and *X*. *oryzae* pathovar, and occasionally between replicates of the same amoebae/bacteria combination; however, the trends remained consistent. In co-cultures with *A*. *castellanii*, bacterial numbers at 24 h did not differ significantly from initial densities, indicating a bacteriostatic effect from the amoebae.

In these studies, the proportion of dead or encysted amoebae was the same over the 24 h experiment (Table 1). About 5% cysts and dead trophozoites were observed at 24 h, but this did not differ from observations during standard culturing phases. *Acanthamoeba* and *Vermamoeba* species generally encyst in adverse conditions, such as extreme temperatures, lack of food or conflict with other microorganisms [34, 35]. The unchanged morphology and viability of the amoebae suggests that *X*. *oryzae* has no deleterious effect on these FLA. Conversely, these data may suggest that the five tested strains of amoebal species were either bacteriostatic or bactericidal to both bacterial pathovars.

**Table 1:** Amoeba with internalized *X*. *oryzae* pv. *oryzae* cells and amoebal morphology after co-culture.

24 h co-cultivation with *X*. *oryzae* pv. *oryzae*					
Amoeba	*A*. *lenticulata*	*A*. *polyphaga*	*A*. *castellanii*	*D*. *discoideum*	*V*. *vermiformis*
With internal bacteria	4.5%	7.5%	0.0%	2.3%	3.3%
Encysted (0h/24h)	6.9/0.6%	8.7/2.5%	3.9/4.1%	2.7/0.0%	5.2/6.1%

### *X*. *oryzae* are rarely consumed by amoebae

**Fig 2:**
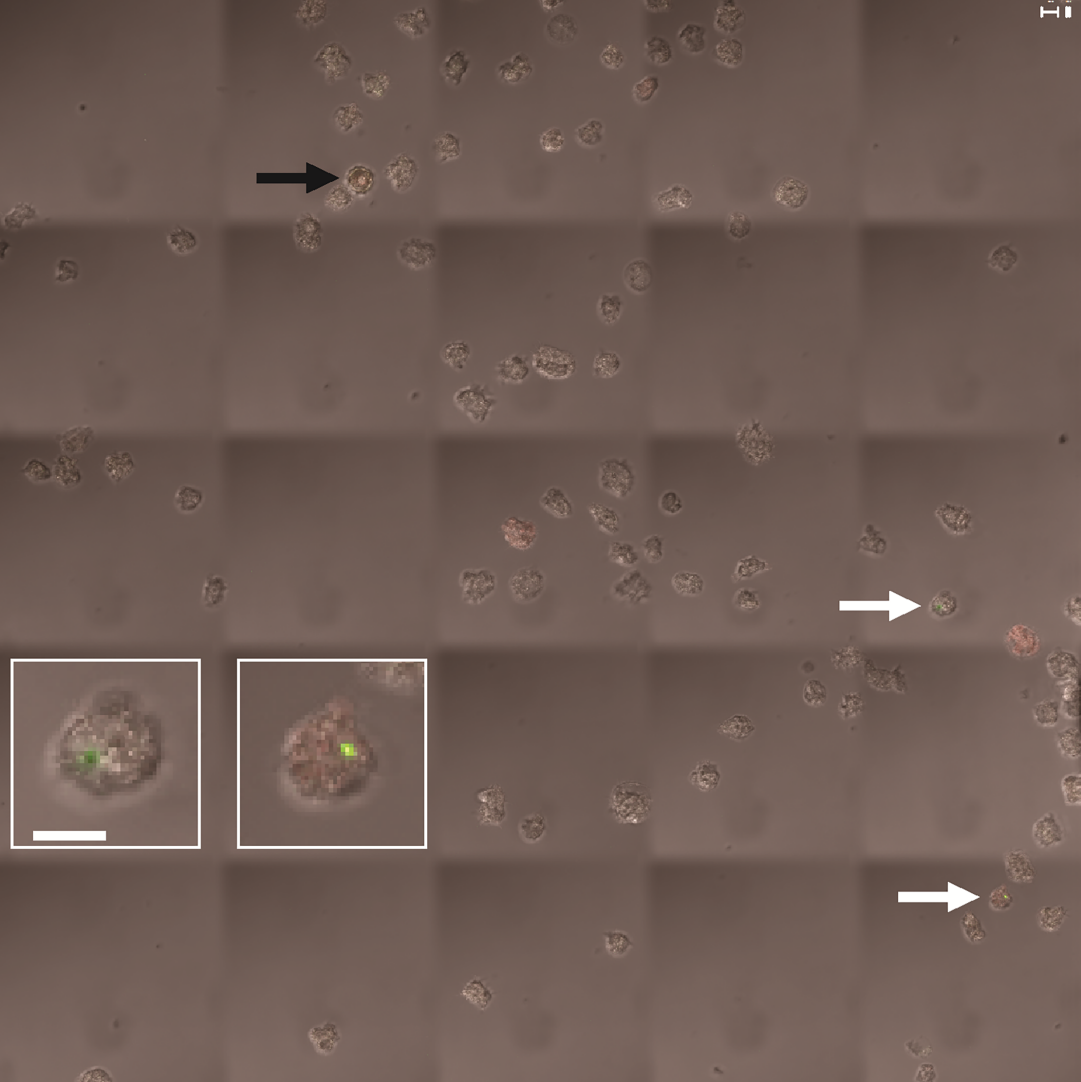
Representative confocal tile scan demonstrating rare internalization of *X*. *oryzae* by amoebae (*A*. *castellanii* depicted). Two instances of internalized bacteria are indicated by white arrows and magnified in the insets. Magnification = 630x, scale bars are 10 μm in length. Merged channels for propidium iodide and Syto9 are shown. Composite image built from replicate scans and processed into a maximum intensity projection by the Zeiss LSM software. White arrows indicate trophozoites with a single Syto9-stained *X*. *oryzae* pv. *oryzae* cell. The presence of bacteria does not force amoebal encystment (cyst indicated by black arrow). For each amoeba/bacteria combination, the experiment was repeated three times, and all experiments had similar results.

Protozoa are known to preferentially feed on gram-negative bacteria, but not all gram-negative bacteria are affected in the same way [5, 36]. In many cases, amoebal trophozoites ingest bacteria as whole cells during feeding [35, 37]. To determine if *X*. *oryzae* are ingested by any of the five amoeba species, at 4 and 24 h after amoebae were incubated with *X*. *oryzae* cells, gentamycin was added to lyse bacteria remaining outside the amoeba (Table 1) [38]. Very few amoebae (< 8%) were observed with internalized *X*. *oryzae* for the three *Acanthamoeba* species, and even fewer for *V*. *vermiformis* (3.3%) and *D*. *discoideum* (2.3%) (Table 1 and Fig 2). The rare bacteria that were observed inside amoebae remained in the cytosol. Viable bacteria were not detected in lysates of amoebae after co-culture with *X*. *oryzae*, suggesting that *X*. *oryzae* does not survive inside amoebae. Stained *X*. *oryzae-*only controls remained fluorescent after over two weeks of incubation and fixation and up to an additional week in storage at 4 °C, demonstrating the lack of signals was not from dye degradation.

The lack of internalized bacteria suggests phagocytosis is not the primary method of bacterial antagonism. The low rates of bacterial consumption were likely not caused by inhibition of amoebal activity or by antagonism of the amoebae by *X*. *oryzae*, because trypan-blue staining did not show amoebal death. Additionally, we did not observe diffuse signals indicative of fluorescent bacteria being digested [37]. The amoebae may be engulfing and rapidly digesting the bacteria, but that scenario is unlikely as bacterial populations were unchanged for the first 4 h of co-culturing in most interactions (Fig 1). In conjunction, these results suggest that amoebae lyse *X*. *oryzae*, but not through phagocytosis. Without internalization, *X*. *oryzae* is not likely capable of using the amoebae as a reservoir to benefit itself, as has been observed for some bacteria [9, 10, 31].

### Amoebae do not degrade *X*. *oryzae* biofilms

**Fig 3:**
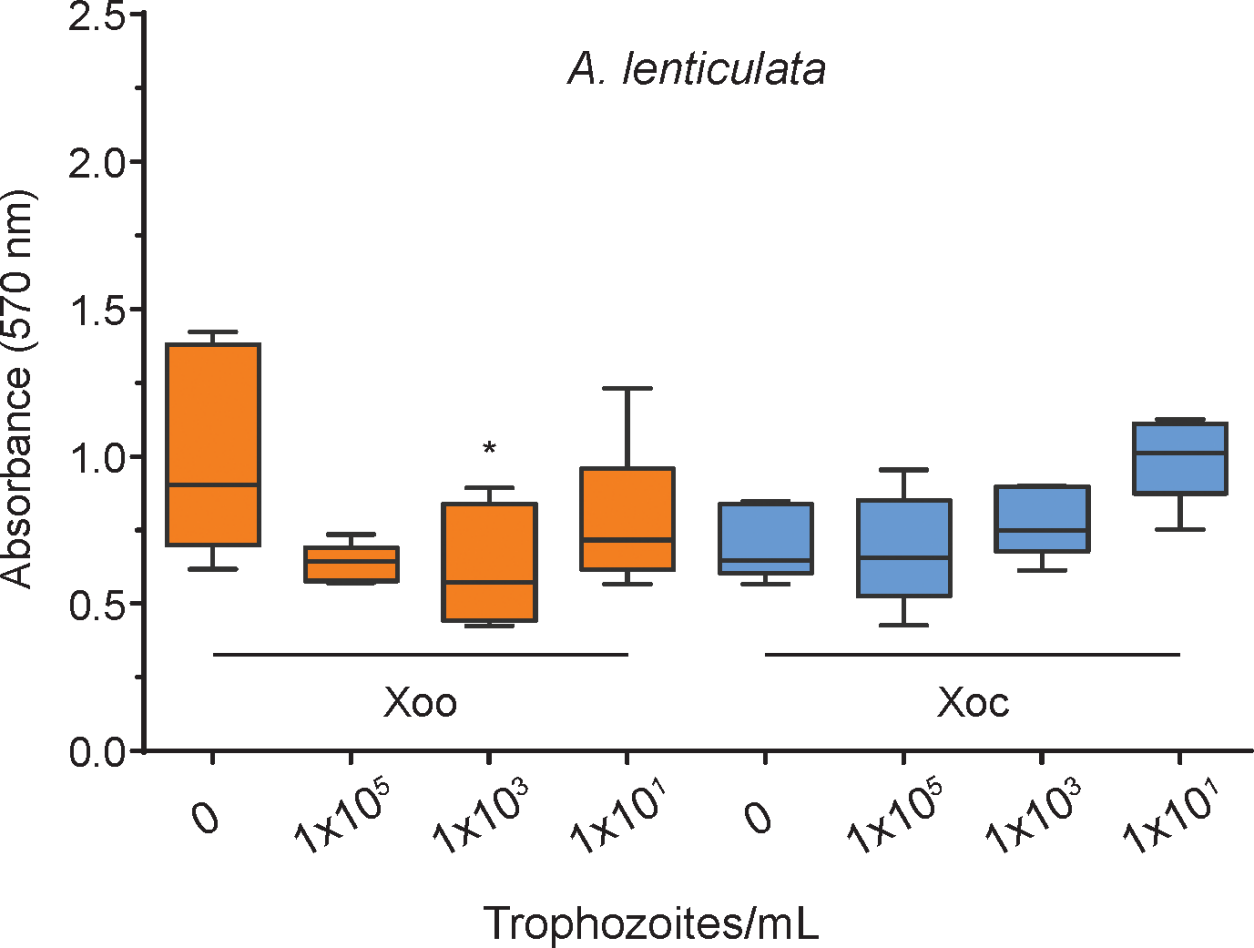
*A*. *lenticulata* trophozoites cause negligible changes in pre-formed *X*. *oryzae* biofilms. Graphs are calculated from six biological replicates per box plot. * denotes p < 0.05, significance calculated using Tukey test in a one-way ANOVA.

*X*. *oryzae* may use a biofilm and exopolysaccharides to prevent predation by FLA [7, 39]. Using crystal violet assays, we found that most amoebae had little to no effect on the integrity of the *X*. *oryzae* biofilms. Of the five amoebae species, only *A*. *lenticulata* significantly degraded biofilms compared to fresh media controls (Fig 3 and S1 Fig).

The other four amoebae species do not reduce biofilm, which suggests that the biofilm serves as a form of protection against amoebal predation (S1 Fig). Future work will be necessary to determine the effect of amoebae inclusion on the quantity of bacteria within these biofilms.

### FLA cell-free supernatants of some FLA are bacteriostatic or bactericidal *X*. *oryzae*

**Fig 4:**
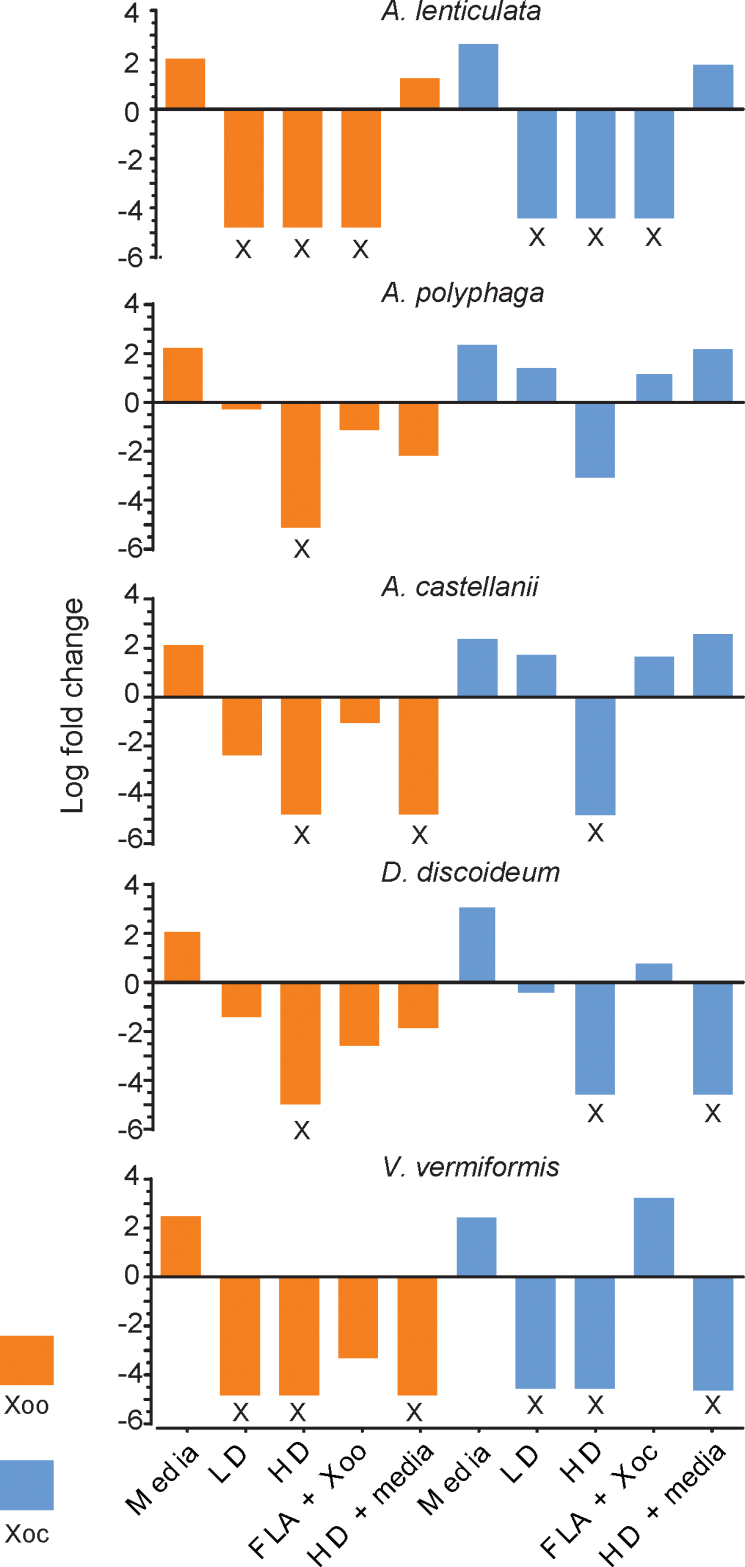
The filtered supernatant from some certain amoeba cultures are either bacteriostatic or bactericidal to *X*. *oryzae*. Representative experiments are presented for each amoeba species: “Media” = medium alone without amoebae; “LD” = low density conditioning culture; “HD” = high density conditioning culture; “FLA + Xoo” = amoebae + *X*. *oryzae* (at a 1:10 ratio); “HD + media” = HD medium supplemented with fresh medium at 1:1. Fold change is calculated as the log(CFU/mL) at 24 h over log(CFU/mL) at 0 h. Each treatment was performed in biological triplicates. Black X’s denote no bacteria could be cultured.

FLA are capable of secreting compounds that lyse and/or kill bacteria [40–42]. To explore the possibility that amoebae release factors capable of killing *X*. *oryzae*, bacterial cells were inoculated into cell-free media previously used to culture amoebae, which we designate as conditioned medium. Media conditioned with low densities of the five amoebae were suppressive or toxic to *X*. *oryzae* cells, because bacteria either multiplied to a lower maximal density or the numbers declined (Fig 4). The suppressive effect was stronger in media conditioned with a high density of amoebae. In nine of the ten amoebae-*X*. *oryzae* combinations, no bacterial colonies were recovered from the high-density conditioned medium. The sole exception was *A*. *polyphaga* with *X*. *oryzae* pv. *oryzicola*. The pronounced effect from the high-density medium suggests that the bactericidal result is related to the concentration of the conditioning culture.

To test if nutrient deprivation in the conditioned media affected bacterial numbers, we included fresh media supplements into the experiment (S1 Table and Fig 4). While conditioned medium from each amoebae species largely had the same effect on both pathovars, only high-density conditioned medium from *A*. *castellanii* and *V*. *vermiformis* remained bactericidal to *X*. *oryzae* pv. *oryzae* after fresh medium was added. In some cases, fresh media supplements to the high-density amoebae conditioned medium diluted or negated the bactericidal effect. The reduced or abolished bactericidal effect from the fresh medium supplement indicates that nutrient deprivation may partially be the cause of harmful effects of *Acanthamoeba* on *X*. *oryzae*.

FLA species may also secrete different antimicrobial factors into their surroundings [40, 42, 43], and the fresh media supplements may have diluted any toxic factor produced by *Acanthamoeba* species, allowing *X*. *oryzae* to survive and multiply. On the other hand, fresh media supplements did not abolish the bactericidal effects from *D*. *discoideum* and *V*. *vermiformis*, suggesting that nutrient deprivation is not a cause for those two species. Additionally, the various amoebae likely secrete unique compounds with different bactericidal activities altogether, and the impact of each is altered to varying degrees by dilution with the fresh-media supplements. Future identification of the factors involved in bacterial toxicity may clarify the differences we observed in conditioned media treatments.

Our results suggest that some amoebae constitutively secrete antibacterial factor(s). In the co-culture assays, bacterial cell density was reduced for most amoeba-bacteria interactions in the first 4 h (Fig 1). To determine if the bactericidal response is stimulated by the presence of *X*. *oryzae*, we included a conditioning treatment of low-density amoebae with *X*. *oryzae* at a ratio of 1:10. Medium conditioned with amoebae and with or without *X*. *oryzae* did not significantly differ from each other (Fig 4 and S2 Fig); therefore, amoeba do not require the presence of *X*. *oryzae* for antimicrobial production.

We also observed bacterial loss at roughly the same rates in the conditioned media assays and co-culture assays. *A*. *lenticulata*, *A*. *polyphaga* and *V*. *vermiformis* significantly reduced bacterial density starting at 4 h in both assays, while effects of *A*. *castellanii* on pv. *oryzicola* were not observed until the 24 h sampling. Responses to *D*. *discoideum* differed between the conditioned media and the co-culture assays. This amoeba specie was bactericidal beginning at 4 h in the conditioned media assays, but had no significant impacts at the same time point in the co-cultures. These discrepancies may have arisen because of different amoebal incubation times and densities, possibly changing how much of the antibacterial agent was produced and secreted. For the other amoebal species, our data show that supernatants from amoebal cultures are sufficient to negatively affect *X*. *oryzae*.

## Conclusion

We identified amoebae species that suppress multiplication or kill cells of two important rice bacterial pathogens, *X*. *oryzae* pvs. *oryzae* and *oryzicola*. The mechanisms for suppression or killing are not due to phagocytosis, but are most likely due to the amoebae’s ability to secrete toxic or inhibitory compounds. Our findings present a previously unknown dynamic between two microorganisms that likely encounter one another in the phytobiome. Future studies should explore the potential of these amoebae as biocontrol agents in the field.

## Supporting information

S1 Table Summary of strains and conditioning cultures. (DOCX)

S1 Fig Crystal violet assays following *X*. *oryzae* biofilm exposure to amoeba trophozoites. Graphs are calculated from a representative experiment with six biological replicates per box plot. * denotes p < 0.05, significance calculated using Tukey test in a one-way ANOVA.
(TIF)

S2 Fig Log cfu/mL of *X*. *oryzae* in conditioned media assays, plotted over time. LD = low density conditioning culture; LD + Xo = conditioning culture with low density amoeba and *X*. *oryzae* at 1:10 ratio; HD = high density conditioning culture; HD + fresh media = HD treatment supplemented with fresh media in a 1:1 mix, final concentration of fresh media supplement equals fresh media only control. ** denotes a p < 0.01 and * denotes p < 0.05 compared to the media-only treatment. Statistical significance tested using two-way ANOVA, Tukey test. N = 4–6 biological replicates.
(TIF)
