# Supplementary material for: Interactions of free-living amoebae with rice bacterial pathogens Xanthomonas oryzae pathovars oryzae and oryzicola
Source: PLoS One. 2018 Aug 24;13(8):e0202941. doi: 10.1371/journal.pone.0202941 (PMC6108499; doi:10.1371/journal.pone.0202941)
Supplement: S1 Table — (DOCX) [file pone.0202941.s001.docx]

| **Organism, strain** | **Original source, source for this manuscript** |
| --- | --- |
| *Acanthamoeba castellanii*, ATCC 30234 | American Type Culture Collection (ATCC; Manassas, VA), WW collection |
| *Acanthamoeba lenticulata*, ATCC 30841 | ATCC, WW collection |
| *Acanthamoeba polyphaga*, Linc-AP1 | ATCC, WW collection |
| *Dictyostelium discoideum*, NC4A1:DBS0236602 | Dicty Stock Center (Chicago, IL), WW collection |
| *Vermamoeba vermiformis*, ATCC 50237 | ATCC, WW collection |
| *Xanthomonas oryzae* pv. *oryzae*, PXO99A | Philippines isolate (Hopkins et al 1992) |
| *Xanthomonas oryzae* pv. *oryzicola*, BLS256 | Philippines (Raymundo & Leach 1993) |

|  | **Conditioned Media Assay Treatments** | | | |
| --- | --- | --- | --- | --- |
|  | **Low density** | **High density** | **High density + fresh media** | **Low density + *X. oryzae*** |
| **Contents during media conditioning** | 1 × 10^5^ amoeba | 1 × 10^6^ amoeba | 1 × 10^6^ amoeba | 1 × 10^5^ amoeba + 1 × 10^6^ *pv. oryzae or oryzicola* |
| **Volume used during *X. oryzae* culturing** | 190 µL | 190 µL | 95 µL + 95 µL of fresh amoeba medium | 190 uL |
