# Supplementary figures and images for: Interactions of free-living amoebae with rice bacterial pathogens Xanthomonas oryzae pathovars oryzae and oryzicola
Source: PLoS One. 2018 Aug 24;13(8):e0202941. doi: 10.1371/journal.pone.0202941 (PMC6108499; doi:10.1371/journal.pone.0202941)

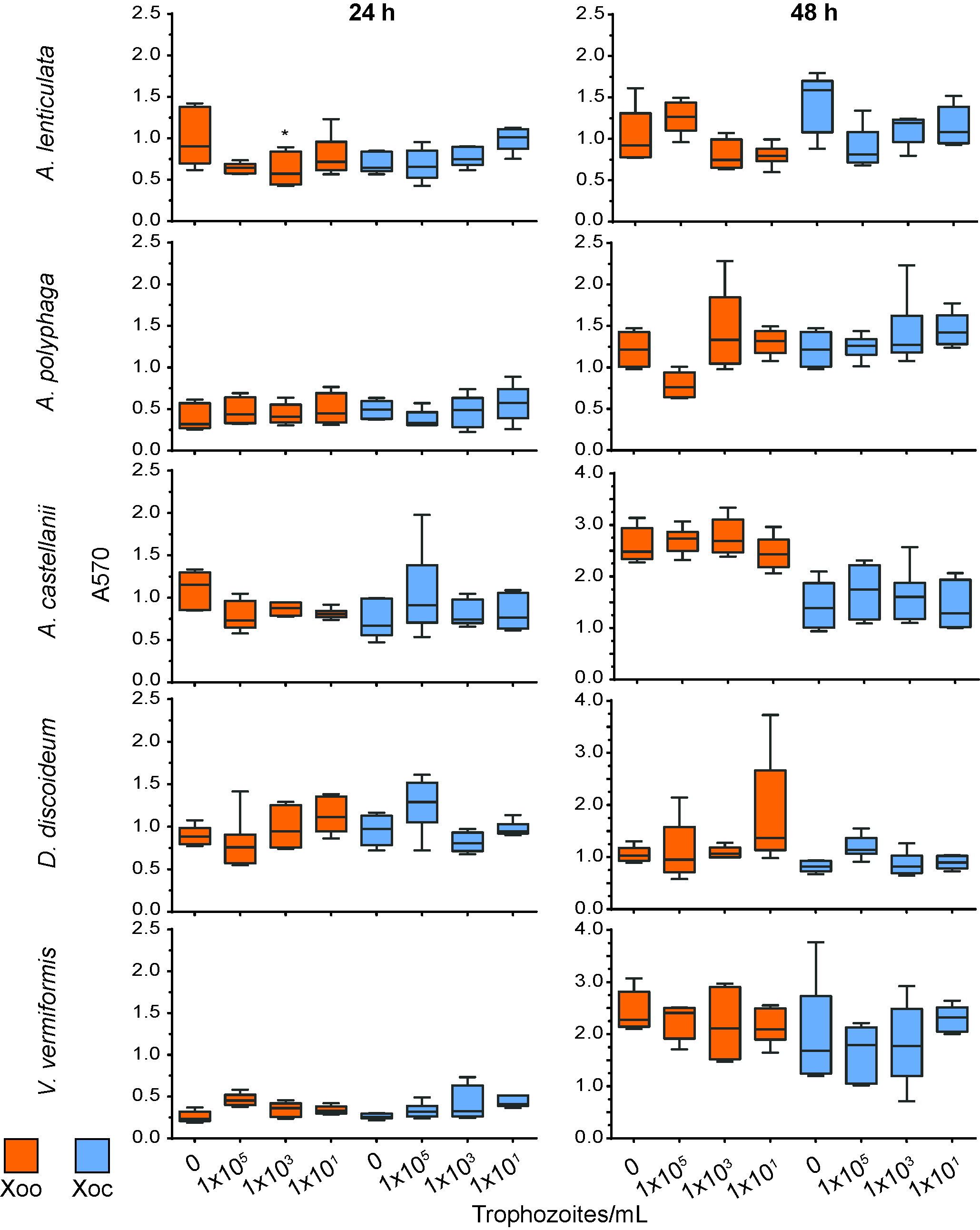

Supplement: S1 Fig — Graphs are calculated from a representative experiment with six biological replicates per box plot. * denotes p < 0.05, significance calculated using Tukey test in a one-way ANOVA. (TIF) [file pone.0202941.s002.tif]

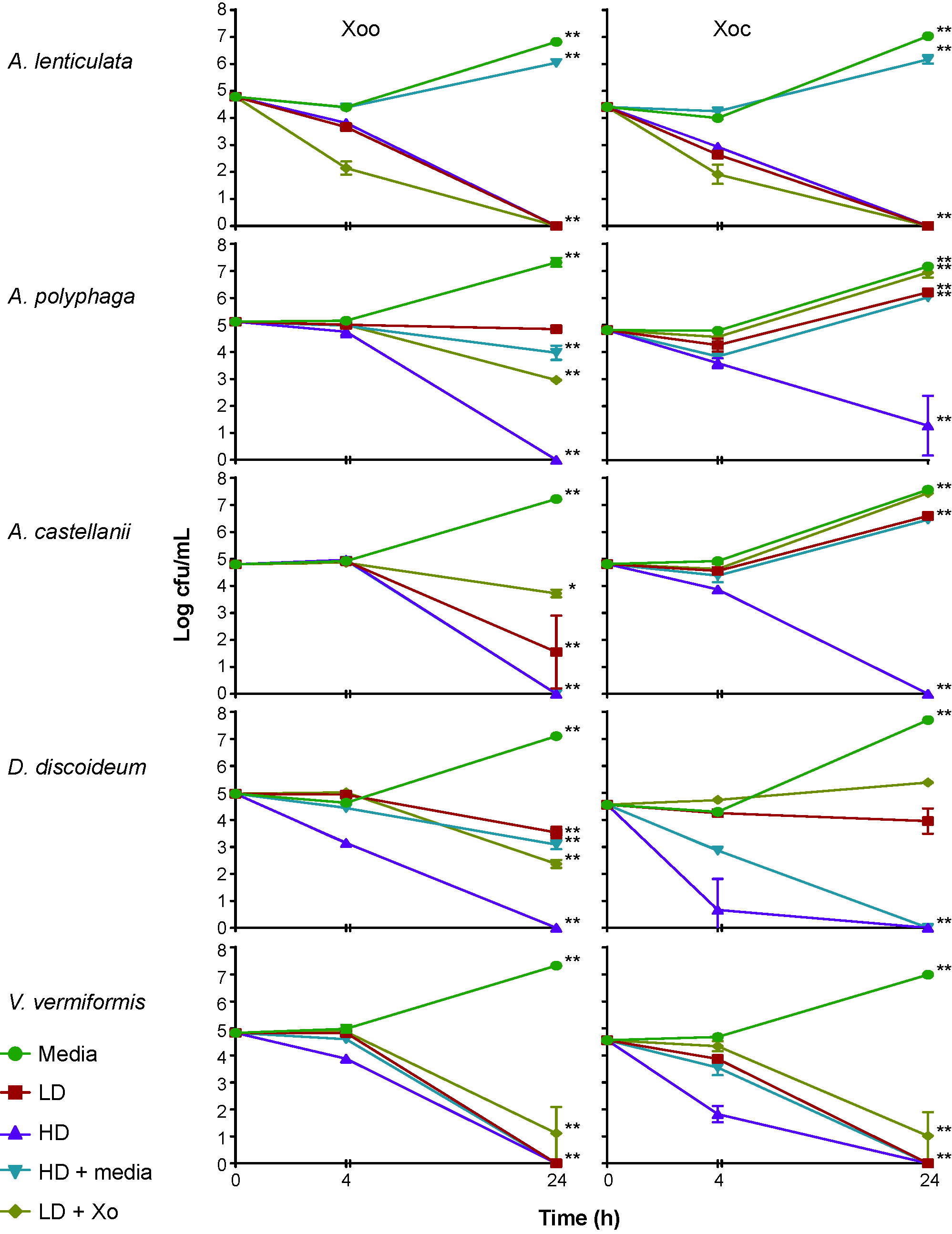

Supplement: S2 Fig — LD = low density conditioning culture; LD + Xo = conditioning culture with low density amoeba and X. oryzae at 1:10 ratio; HD = high density conditioning culture; HD + fresh media = HD treatment supplemented with fresh media in a 1:1 mix, final concentration of fresh media supplement equals fresh media only control. ** denotes a p < 0.01 and * denotes p < 0.05 compared to the media-only treatment. Statistical significance tested using two-way ANOVA, Tukey test. N = 4–6 biological replicates. (TIF) [file pone.0202941.s003.tif]
